# Evaluation of SARS‐CoV‐2 neutralizing antibodies using a CPE‐based colorimetric live virus micro‐neutralization assay in human serum samples

**DOI:** 10.1002/jmv.25986

**Published:** 2020-05-17

**Authors:** Alessandro Manenti, Marta Maggetti, Elisa Casa, Donata Martinuzzi, Alessandro Torelli, Claudia M. Trombetta, Serena Marchi, Emanuele Montomoli

**Affiliations:** ^1^ VisMederi Research s.r.l. Siena Italy; ^2^ VisMederi s.r.l. Siena Italy; ^3^ Department of Molecular and Developmental Medicine University of Siena Siena Italy

**Keywords:** epidemiology, humoral immunity, neutralization, pandemic, SARS coronavirus

## Abstract

The micro‐neutralization assay is a fundamental test in virology, immunology, vaccine assessment, and epidemiology studies. Since the SARS‐CoV‐2 outbreak at the end of December 2019 in China, it has become extremely important to have well‐established and validated diagnostic and serological assays for this new emerging virus. Here, we present a micro‐neutralization assay with the use of SARS‐CoV‐2 wild type virus with two different methods of read‐out. We evaluated the performance of this assay using human serum samples taken from an Italian seroepidemiological study being performed at the University of Siena, along with the human monoclonal antibody CR3022 and some iper‐immune animal serum samples against Influenza and Adenovirus strains. The same panel of human samples have been previously tested in enzyme‐linked immunosorbent assay (ELISA) as a pre‐screening. Positive, borderline, and negative ELISA samples were evaluated in neutralization assay using two different methods of read‐out: subjective (by means of an inverted optical microscope) and objective (by means of a spectrophotometer). Our findings suggest that at least 50% of positive ELISA samples are positive in neutralization as well, and that method is able to quantify different antibody concentrations in a specific manner. Taken together, our results confirm that the colorimetric cytopathic effect‐based microneutralization assay could be used as a valid clinical test method for epidemiological and vaccine studies.

## Highlights

- High sensitivity of the Micro‐Neutralization assay for anti‐SARS‐CoV‐2 neutralizing antibodies detection in serum samples.
- CPE‐based (Subjective) and Colorimetric‐based (Objective) Micro‐Neutralization read out are comparable in terms of results provided.
- 50% of S1 ELISA positive serum samples present neutralizing antibodies against wild type SARS‐CoV‐2 virus.

## 1 INTRODUCTION

Coronavirus (CoV), along with Influenza virus, is a major public health concern. CoVs are enveloped, positive single‐stranded RNA viruses belonging to the *Coronaviridae* family; they contain a single genome of 30 Kbp, and consist of four groups: *Alphacoronavirus*, *Betacoronavirus*, *Gammacoronavirus*, and *Deltacoronavirus*.^1^, ^2^ To date, seven CoV strains are known to infect humans, affecting the lower respiratory tract, gastrointestinal system, heart, liver, kidney, and central nervous system.^3^, ^4^ Over the past 23 years, outbreaks in humans, including Severe Acute Respiratory Syndrome (SARS) and Middle‐East Respiratory Syndrome (MERS),^5^ have heightened the daunting possibility that a future pandemic may be caused by one of these agents, underlining the urgent need to prepare for such an eventuality, since no vaccines or approved therapies, are as yet available.^6^ At the end of December 2019 in Wuhan, Hubei Province, China, a novel CoV strain, called SARS‐CoV‐2 by the International Committee on Taxonomy of Viruses (ICTV), caused 27 cases of pneumonia of unidentified etiology.^7^ Due to the rapid and uncontrollable spread of the virus in almost every country in the world, the World Health Organization (WHO) officially declared the pandemic status in March 2020. The disease caused by SARS‐CoV‐2, named COVID‐19, is considered a self‐limiting infectious disease with five different possible outcomes: asymptomatic cases (1.2%), mild cases (80.9%), severe cases (13.8%), critical cases (4.7%), and deaths (2.3%).^7^, ^8^ However, some authors reported a higher percentage of asymptomatic infections in children under the age of 10 (15.8%).^9^ Because of the lack of specific antiviral drugs or vaccines, several thousands of serious cases and deaths occur every day all over the world, and strict quarantine measures have been imposed either nationally or internationally. Since the antibody response of the serum, after a natural CoV infection remains detectable for a long time,^10^ medical authorities in many countries are trying to calculate the percentage of the population that may be protected against the new circulating strain through the assessment of anti‐SARS‐CoV‐2 Immunoglobulin G (IgG) and M (IgM) levels in serum samples. Principal serological tests used in these studies are ELISA‐based assays. Most of these tests focus on different combinations of coatings on the viral spike (S) protein (*S1; S1*+*S2; S1‐S2 extracellular domain*‐*ECD*, *receptor binding domain*‐RBD), due to the fact that the CoV's ability to attach and consequently enter the cell is mainly mediated by this protein.^11^ Enzyme‐linked immunosorbent assays (ELISAs) certainly have advantages, such as high throughput, speed of testing, and the possibility of avoiding the requirement for a high containment laboratory, as BSL 3. However, most of these assays present some limitations, such as low specificity and sensitivity, and use of alternative purified proteins that can be produced in different hosts (human‐derived cells vs insect cells). In addition, the mismatch between results obtained from the same samples, using different ELISA reagents and coatings (eg, source of antigen), may lead to confusion.^12^ To date, the Micro‐Neutralization assay (MN), currently considered the gold‐standard is the most specific and sensitive serological assay capable of evaluating and detecting, functional neutralizing antibodies (nAbs). In this paper, a live virus‐based MN assay is presented for the quantification of SARS‐CoV‐2‐specific nAbs in human serum samples by two different methods of detection: a classical read‐out by checking the percentage of cytopathic effect (CPE) in the cell monolayer, and a colorimetric read‐out by a spectrophotometer.

## 2 MATERIALS AND METHODS

### 2.1 Serum samples and human monoclonal antibody IgG1

A total of 83 human serum samples were collected as part of a seroepidemiological study that is being performed in the laboratory of Molecular Epidemiology of the University of Siena, Italy. Serum samples were anonymously collected in compliance with Italian ethics law. The human monoclonal antibody IgG1‐*CR3022* (absolute antibody) was tested along with the serum samples in the MN assay and ELISA. Hyperimmune sheep antisera against Influenza A/H1N1/California/7/2009 (10/218), B/Brisbane/60/2008 (13/312), and A/Anhui/1/2013 (15/248) strains were purchased from the National Institute for Biological Standard and Controls (NIBSC, UK). Hyperimmune rabbit serum samples against Adenovirus Type 4 (V204‐502‐565) were provided by the National Institute of Allergy and Infectious Diseases (NIH, Bethesda). Human serum minus IgA/IgM/IgG (S5393‐1VL) (Sigma, St. Louis, MO) was used as a negative control.

### 2.2 Cell culture

VERO cells, an African Green monkey kidney cell line, were purchased from the European Collection of Authenticated Cell Cultures (ECACC ‐ Code 84121903). VERO cells were cultured in Eagle's minimum essential medium (EMEM) (Lonza, Milano, Italy) supplemented with 2 mM L‐ Glutamine (Lonza, Milano, Italy), 100 units/mL penicillin‐streptomycin mixture (Lonza, Milano, Italy) and fetal bovine serum (FBS) (Euroclone, Pero, Italy) to a final concentration of 5%, at 37°C, in a 5% CO_2_ humidified incubator.

VEROE6 cells, an epithelial cell line from the kidney of a normal monkey (*Cercopithecus aethiops*), were acquired from the American Type Culture Collection (ATCC ‐ CRL 1586).

Huh‐7 cells, an epithelial cell line from Human hepatocellular carcinoma, were kindly provided by the University of Siena (ECACC‐Code 01042712). Both VEROE6 and Huh‐7 cells were cultured in Dulbecco's Modified Eagle's Medium (DMEM)‐high glucose (Euroclone, Pero, Italy) supplemented with 2 mM l‐Glutamine (Lonza, Milano, Italy), 100 units/mL penicillin‐streptomycin mixture (Lonza, Milano, Italy) and 10% of FBS, at 37°C, in a 5% CO_2_ humidified incubator.

Adherent sub‐confluent cell monolayers of VERO, VERO E6, and Huh‐7 were prepared in growth medium, E‐MEM or D‐MEM high glucose containing 2% FBS in T175 flasks or 96‐well plates for propagation or titration and neutralization tests of SARS‐CoV‐2, respectively.

### 2.3 Virus and titration

SARS CoV‐2 2019‐*2019‐nCoV strain 2019‐nCov/Italy‐INMI1*‐wild type virus was purchased from the European Virus Archive goes Global (EVAg, Spallanzani Institute, Rome). The virus was titrated in serial 1 log dilutions (from 1 log to 11 log) to obtain a 50% tissue culture infective dose (TCID50) on 96‐well culture plates of VERO and VERO E6 cells. The plates were observed daily for a total of 4 days for the presence of CPE by means of an inverted optical microscope. The end‐point titres were calculated according to the Reed & Muench method^13^ based on eight replicates for titration.

### 2.4 Viral growth in cell culture

The SARS‐CoV‐2 virus was seeded and propagated in VERO, VERO E6, and Huh‐7 cells by using EMEM (for VERO and Huh‐7) and DMEM high glucose (for VERO E6) both supplemented with 2% FBS and 100 IU/mL penicillin‐streptomycin.

Cells were seeded in T175 flasks at a density of 1 × 10^6^ cells/mL. After 18 to 20 hours, the sub‐confluent cell monolayer was washed twice with sterile Dulbeccos's phosphate buffered saline (DPBS). After removal of the DPBS, the cells were infected with 3.5 mL of EMEM/DMEM 2% FBS containing the virus at a multiplicity of infection of 0.001 and 0.01. After 1 hour of incubation at 37°C in a humidified atmosphere with 5% CO_2_, 50 mL of EMEM/DMEM containing 2% FBS was added for VERO‐Huh7/VERO E6. The flasks were daily observed and the virus was harvested when 80%‐90% of the cells manifested CPE. The culture medium was centrifuged at +4°C 1600 rpm for 8 minutes, to remove the cell debris, then they aliquoted and stored at −80°C.

### 2.5 Micro‐neutralization assay

Serum samples were heat‐inactivated for 30 minutes at 56°C; two‐fold serial dilutions, starting from 1:10, were then mixed with an equal volume of viral solution containing 100 TCID50 of SARS‐CoV‐2. The serum‐virus mixture was incubated for 1 hour at 37°C in a humidified atmosphere with 5% CO_2_. After incubation, 100 µL of the mixture at each dilution was added in duplicate to a cell plate containing a semi‐confluent VERO E6 monolayer. The plates were incubated for 4 days at 37°C in a humidified atmosphere with 5% CO_2_.

#### 2.5.1 CPE‐read out

After 4 days of incubation, the plates were inspected by an inverted optical microscope. The highest serum dilution that protected more than the 50% of cells from CPE was taken as the neutralization titre.

#### 2.5.2 Colorimetric read‐out

After 3 days of incubation, the supernatant of each plate was carefully discarded and 100 µl of a sterile DPBS solution containing 0.02% neutral red (Sigma, St. Louis, MO) was added to each well of the MN plates. After 1 hour of incubation at room temperature, the neutral red solution was discarded and the cell monolayer was washed twice with sterile DPBS containing 0.05% Tween 20. After the second incubation, the DPBS was carefully removed from each well; then, 100 µL of a lysis solution made up of 50 parts of absolute ethanol (Sigma, St. Louis, MO), 49 parts of MilliQ and 1 part of glacial acetic acid (Sigma) was added to each well. Plates were incubated for 15 minutes at room temperature and then read by a spectrophotometer at 540 nm. The highest serum dilution, showing an optical density (OD) value greater than the cut‐off value, was considered as the neutralization titre. The cut‐off value is calculated as the average of the OD values of the cell control wells divided by two.

### 2.6 Enzyme‐linked immunosorbent assay

Specific anti‐SARS‐CoV‐2 IgG antibodies were detected through a commercial ELISA kit (Euroimmun, Lübeck, Germany). ELISA plates are coated with recombinant structural protein (S1 domain) of SARS‐CoV‐2. According to the manufacturer, cross‐reactions may occur with anti‐SARS‐CoV(‐1) IgG antibodies, due to the close relationship between SARS‐CoV(‐1) and SARS‐CoV‐2, while cross‐reactions with other human pathogenic CoVs (MERS‐CoV, HCoV‐229E, HCoV‐NL63, HCoV‐HKU1, and HCoV‐OC43) are excluded. The assay provides semi‐quantitative results by calculating the ratio of the OD of the serum sample over the OD of the calibrator. According to the manufacturer's instructions, positive samples have a ratio ≥1.1, borderline samples a ratio between 0.8 and 1.1 and negative samples a ratio <0.8.

### 2.7 Statistics analysis

Data analysis was performed using GraphPad Prism Version 5 and Microsoft Excel 2019. Friedman test was used to compare viral titres obtained at different time points during viral growth in cell culture. A *P* value <.05 was considered statistically significant.

## 3 RESULTS

### 3.1 High viral load for VERO and VERO E6, no propagation for Huh‐7

**Figure 1:**
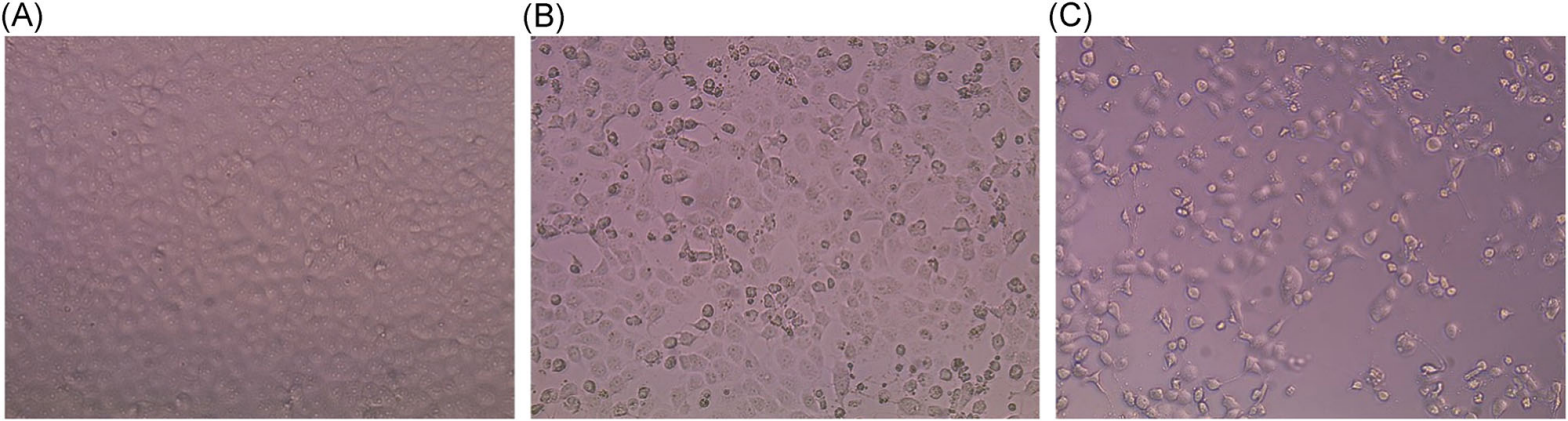
Vero E6 cells at different stage of infection. A, Not infected VERO E6 cell monolayer after 72 hours, complete absence of CPE. B, SARS‐CoV‐2 infected VERO E6 cell monolayer after 36 hours postinfection, 20%‐30% of CPE recovered. C, SARS‐CoV‐2 infected VERO E6 after 52 hours postinfection, 80% of CPE recovered. CPE, cytopathic effect; SARS‐CoV‐2, Severe Acute Respiratory Syndrome‐Coronavirus‐2

**Figure 2:**
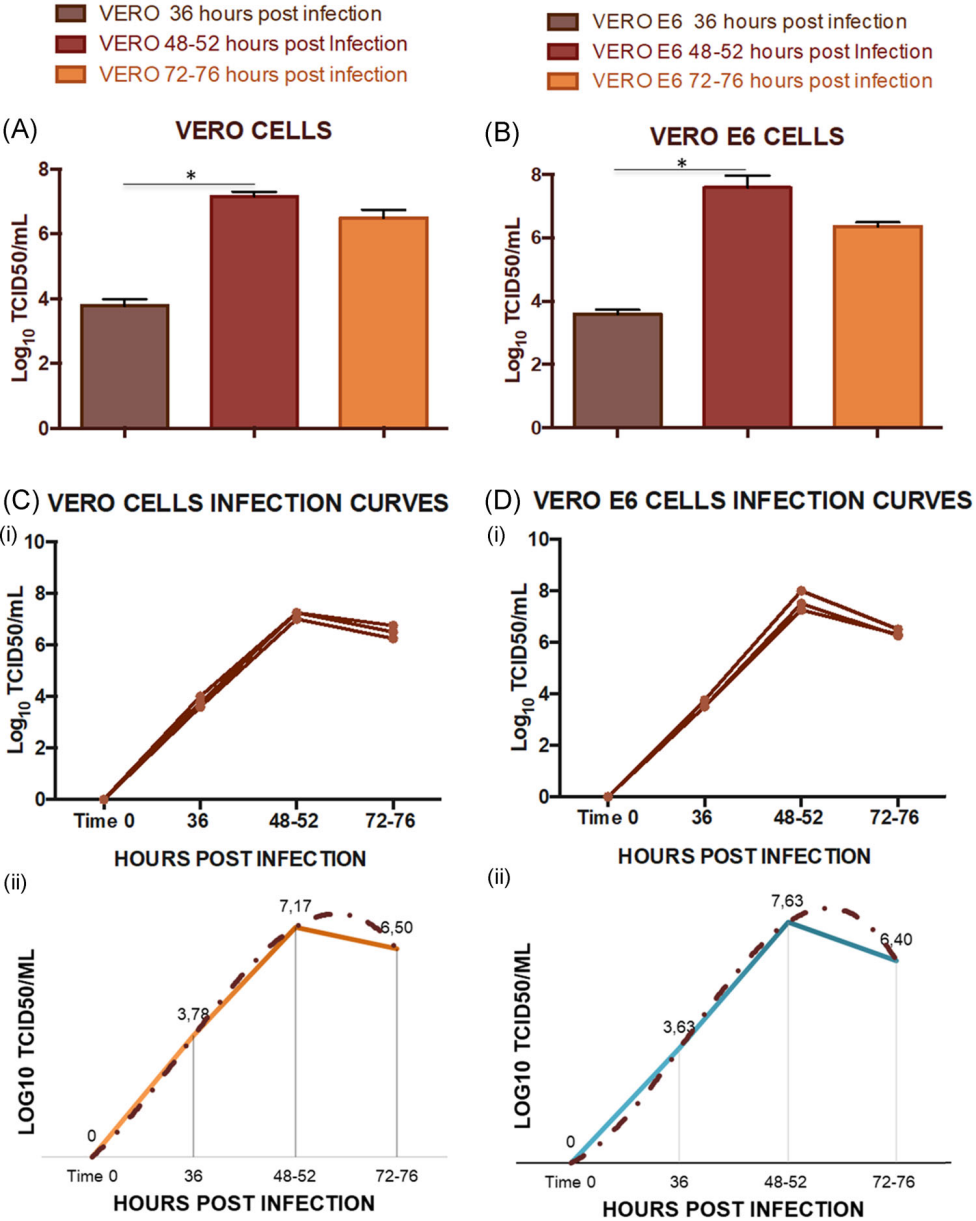
Viral titres reached for VERO and VERO E6 in three different viral infection experiments in T‐175 flasks. A, Titres registered in triplicate (n = 3) for VERO cells after 36, 48 to 52 and 72 to 76 hours post infection. A significant increase in the viral titre has been registered after 48 to 52 hours according to Friedman test (*P* < .05), error bars indicate the standard deviation among the three independent measures. B, Titres registered (n = 3) for VERO E6 cells after 36, 48 to 52 and 72 to 76 hours post infection. A significant increase in the viral titre has been registered after 48 to 52 hours according to Friedman test (*P* < .05), error bars indicate the standard deviation among the three independent measures. C.1, Infection curves for VERO cells for three independent experiments of viral growth. C.2, Polynomial infection curve derived from the average of the three experimental curves for VERO cells. D.1, Infection curves for VERO E6 for three independent experiments of viral growth. D.2, Polynomial infection curve derived from the average of the three experimental curves for VERO E6 cells

SARS‐CoV‐2 has been propagated for three times in three independent experiments in VERO, VERO E6, and Huh‐7 cells. We decided to investigate the viral growth in these specific cell lines because of, as reported in literature, they are the preferred lines for SARS‐CoV isolation and replication.^14^, ^15^ Different harvest time‐points were evaluated to obtain the infection curve for each cell line: 36, 48 to 52 and 72 to 76 hours postinfection. A high viral titre was obtained for VERO and VERO E6 cells. In both cell lines we tried two different multiplicity of infection (MOI) (0.001 and 0.01), starting from a viral stock containing 10^7.25^ TCID50/mL (only results for MOI = 0.001 are reported in this study). After 24 hours postinfection, no CPE or infection plaques were observed in the cell monolayer in any of the three cell lines. After 36 hours, VERO E6 and VERO T‐Flasks proved to have detectable CPE of 30%‐40% (10^3.63^ TCID50/mL ± 0.14 SD) and 15%‐20% (10^3.78^ TCID50/mL ± 0.2 SD), respectively. Between 48 and 52 hours after infection, both cell lines reached 80% of CPE (Figure 1) recording a significant increase of the viral titre according to Friedman test with a mean equal to 10^7,63^ TCID50/mL ± 0.38 SD for VERO E6 cells, and 10^7.17^ TCID50/mL ± 0.1 SD for VERO cells. Lower titres were registered in flasks 72 to 76 hours postinfection for VERO (10^6.5^ TCID50/mL ± 0.2 SD) and VERO E6 (10^6.4^ TCID50/mL ± 0.13 SD), with flasks showing 100% of CPE (Figure 2). No detectable CPE was observed for Huh‐7 cells up to the 7th day after infection.

To check the viral production in Huh‐7 cells, we passed the supernatant in VERO E6 cells but no CPE was detected in this cell line. This confirms that Huh‐7 cells are not able to support the viral replication of this CoV strain, as already showed by Harcourt et al.^16^ The supernatants derived from VERO, VERO E6 and Huh‐7 were titrated in 96‐well plates, which were read after 72 hours; titres reached ranged from 10^6.2^ to 10^7.8^ TCID50/mL either for VERO and VERO E6‐derived virus; no titre has been detected for Huh‐7‐derived virus (data not shown).

### 3.2 Comparison between ELISA and MN assays

A total of 83 serum samples were tested for the presence of anti‐SARS‐CoV‐2 antibodies by ELISA and MN assay. On ELISA, 42 samples proved positive, 20 borderline and the remaining 21 negative. Along with the human serum samples, to evaluate the specificity of the MN assay, we tested several animal sera that were highly immunized against different viral diseases, such as Influenza (seasonal and pandemic) and Adenovirus type 4. These sera proved to have high nAb titres against the homologous strain in the MN assay (data not shown). In the MN assay, we assessed the serum response by using two different viral infective doses: a standard dose of 100 TCID50/well and a lower dose of 25 TCID50/well. Neutralization test results confirmed the complete absence (100%) of nAbs in samples already negative on ELISA. Of the 42 samples positive on ELISA, 22 (52.3%) confirmed the presence of CPE‐inhibiting nAbs in the cell monolayer, with titres ranging between 10 and 1280/2560. Of 20 borderline ELISA samples, only 3 (15%) confirmed the capability of neutralizing the virus on MN assay. Each sample was tested in duplicate by two different operators, to confirm and validate the results obtained. Each sample was also evaluated by the colorimetric read‐out. The results yielded by MN on using the lower infective dose (25 TCID50) were in line with those obtained with the standard infective dose; in some cases, however, we detected a titre that was one dilution step higher, which maintained all negative sample negative (Table 1). All animal samples tested against Influenza and Adenovirus type 4 proved completely negative, confirming the specificity of the MN assay in the detection of anti‐SARS‐CoV‐2 nAbs.

**Table 1:** ELISA and neutralization results for all 83 human serum samples

Sample ID	ELISA	MN CPE titre analyst 1 100 TCID_50_	MN CPE titre analyst 2 100 TCID_50_	Colorimetric MN 100 TCID_50_	MN CPE titre 25 TCID_50_
From 1 to 21	Negative	5	5	5	5
22	Borderline	5	5	5	5
23	Borderline	5	5	5	5
24	Borderline	5	5	5	5
25	Borderline	5	5	5	5
26	Borderline	5	5	5	5
27	Borderline	5	5	5	5
28	Borderline	5	5	5	5
29	Borderline	5	5	5	5
30	Borderline	5	5	5	5
31	Borderline	5	5	5	5
32	Borderline	5	5	5	5
33	Borderline	5	5	5	5
34	Borderline	5	5	5	5
35	Borderline	5	5	5	5
36	Borderline	5	5	5	5
37	Borderline	5	5	5	5
38	Borderline	5	5	5	5
22	Borderline	20	20	20	40
23	Borderline	80	40	80	80
24	Borderline	20	20	20	20
42	Positive	640	640	640	640
43	Positive	20	20	20	40
44	Positive	320	320	320	320
45	Positive	640	320	320	640
46	Positive	40	40	40	40
47	Positive	640	640	640	640
48	Positive	20	20	20	20
49	Positive	10	20	10	20
50	Positive	160	320	320	320
51	Positive	40	40	40	40
52	Positive	160	160	160	320
53	Positive	640	640	640	640
54	Positive	80	80	80	80
55	Positive	1280	2560	1280	1280
56	Positive	160	160	160	320
57	Positive	80	80	80	80
58	Positive	10	10	10	20
59	Positive	80	80	80	80
60	Positive	640	640	640	640
61	Positive	10	10	10	10
62	Positive	40	40	40	40
63	Positive	40	40	40	40
64	Positive	5	5	5	5
65	Positive	5	5	5	5
66	Positive	5	5	5	5
67	Positive	5	5	5	5
68	Positive	5	5	5	5
69	Positive	5	5	5	5
70	Positive	5	5	5	5
71	Positive	5	5	5	5
72	Positive	5	5	5	5
73	Positive	5	5	5	5
74	Positive	5	5	5	5
75	Positive	5	5	5	5
76	Positive	5	5	5	5
77	Positive	5	5	5	5
78	Positive	5	5	5	5
79	Positive	5	5	5	5
80	Positive	5	5	5	5
81	Positive	5	5	5	5
82	Positive	5	5	5	5
83	Positive	5	5	5	5

*Note*: Negative samples are indicated in the first row of the table. Neutralizing titres, obtained with CPE (100 and 25 TCID50 infective dose) and colorimetric read‐out methods, are indicated for each sample.

### 3.3 Absence of neutralizing activity for human IgG1 monoclonal antibody CR3022

As reported^17^ that the *CR3022* monoclonal antibody (mAb) has a high capability of neutralizing the SARS‐CoV strain, we included this mAb (IgG1) within the human serum samples in our neutralization assay. The CR3022 antibody targets a highly conserved epitope on the RBD of SARS‐CoV. The concentrations tested in MN ranged from 10 µg down to 0.009 µg. The monoclonal antibody was pre‐incubated for 1 hour with 100 TCID50 of live SARS‐CoV‐2 virus before being passed on the VERO E6 monolayer. After 72 hours of incubation, no neutralizing activity was obtained at any of the concentrations tested. By contrast, very high ELISA titres were detected (data not shown). As reported by Tian et al,^18^ CR3022, unlike other SARS‐CoV monoclonal antibodies, recognizes a different epitope from that one recognized on the RBD by the ACE2 receptor. Moreover, the C‐terminal RBD residue of SARS‐CoV‐2 virus has been found to be quite different from that of SARS‐CoV, which may have a critical impact on the cross‐reactivity of neutralizing antibodies. Also, as already reported by Tian et al,^19^ some antibodies with a high capability of neutralizing SARS‐CoV, were found to be unable to bind the S protein of the new SARS‐CoV‐2 strain; this requires new dedicated monoclonal antibodies.

### 3.4 Neutralization assay read‐out: subjective vs objective methods

**Figure 3:**
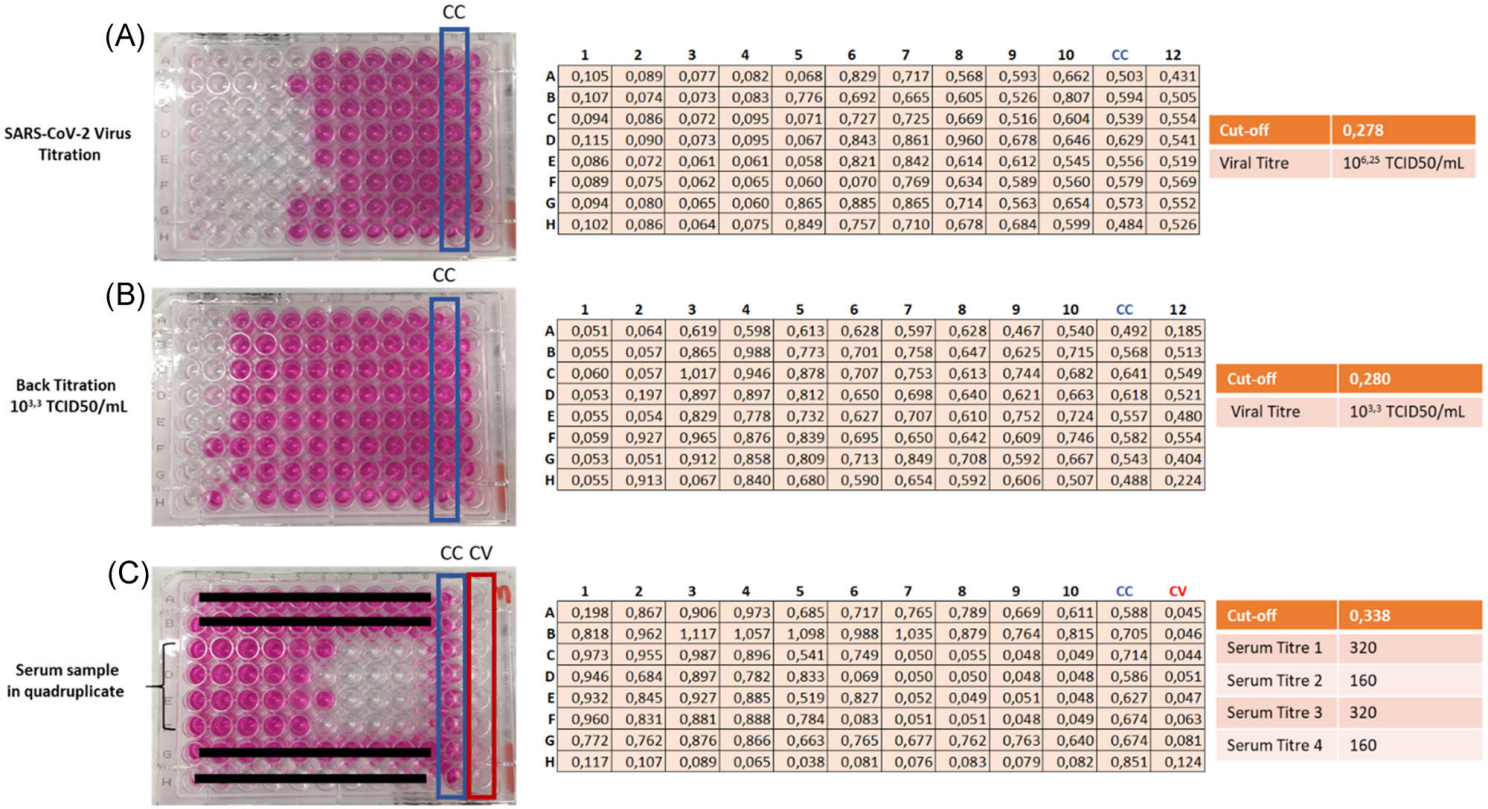
Schematic overview of the colorimetric MN read‐out. A, SARS‐CoV‐2 virus titration. B, Titration of the working viral solution. C, Neutralization plate with a serum sample tested in quadruplicate. In each plate, the column highlighted in blue is the cell control (highest OD value), while the column highlighted in red is the virus control (no OD values). The cut‐off value is evaluated for each plate, and is equal to the average of the cell control ODs divided by two. Wells that show OD values lower than the cut‐off are considered virus‐positive, and hence infected. The viral titres in both the stock solution (A) and the working viral solution (B) are calculated by means of the Reed and Muench method. The titre of the serum sample (C) was calculated as the reciprocal of the highest dilution at which the OD value was higher than or equal to the cut‐off value. OD, optical density; ARS‐CoV‐2, Severe Acute Respiratory Syndrome‐Coronavirus‐2

The results obtained in the MN assay in all serum samples were evaluated through two methods of read‐out: by inspecting the inhibition of the CPE at each serum dilution (subjective method) by an inverted optical microscope, and by applying a colorimetric method in which the healthy cell monolayer is stained with a neutral red solution. As can be seen in Figure 3, the 12th and the 11th columns of each plate were set up as a virus control (CV) and a cell control (CC), respectively. Serum samples were progressively diluted from column 1 to column 10. The cut‐off value, calculated mathematically as the average of all cell control ODs divided by two, indicates the titre of each sample tested. Results of the comparison between ELISA and MN (Table 1) suggest that a well‐trained operator is able to read the CPE, thereby providing the same results as the spectrophotometer in terms of titre with no differences between the results provided by the two different operators and the spectrophotometric evaluation of the ODs.

One of the advantages of the colorimetric read‐out is that, being a completely automated method, it offers a higher throughput, while inspection of each dilution well by means of the optical microscope slows down the process.

## 4 DISCUSSION

The availability of a specific serological assay capable of providing the most reliable and accurate antibody response in a given sample is a crucial factor in all epidemiological studies. This is particularly important in an emergency situation, such as during a sudden epidemic or, even worse, a pandemic. Indeed, knowing which percentage of the population has already come in contact with the virus, and consequently developed a specific immune response, can drive the type and timing of prevention and containment measures. Virus nAbs can be induced by natural infection or vaccination, and they have a crucial role in controlling and limiting viral infection and transmission among people. In this paper, we present a possible approach to evaluate anti‐SARS‐CoV‐2 neutralizing antibodies in human and animal samples using the wild‐type virus. We evaluated the performance of the MN assay on a subset of samples that are being tested by ELISA in a seroepidemiological study currently underway at the University of Siena. We also tested four animal antisera against Influenza and Adenovirus and human CR3022 mAb. Since SARS‐CoV‐2 and SARS‐CoV display a high sequence identity of the S protein,^18^ it is possible that SARS‐CoV nAbs may elicit cross‐neutralization activity against SARS‐CoV‐2. Unfortunately, our preliminary neutralization results showed no ability of the CR3022 mAb to prevent viral attachment and entry into cell monolayer, which developed CPE in less than 48 hours postinfection. On the other hand, the high signal registered on ELISA confirmed the potential of the CR3022 mAb to bind with high affinity an epitope on the RBD of the SARS‐CoV‐2 S protein.^19^ For human serum samples, the MN assay confirmed that at least 50% of the samples, tested positive on ELISA assay, presented antibodies with neutralizing ability. This finding is broadly in line with previous Influenza studies, in which that assay was able to detect all binding antibodies without a prediction of their functionality.^20^, ^21^ It is interesting to note that the ELISA kit used in the present study has been validated for sensitivity and specificity for SARS‐CoV‐2 by Okba et al in a previous work,^22^ and it has been found to have 96% of specificity and 65% of sensitivity compared to other 8 commercial ELISA kits for SARS‐CoV‐2.^23^ The fact that we detected fairly low neutralizing titres in samples and that only half of those assessed positive on ELISA may be due to different factors: (a) at this stage the human population is completely naïve about this CoV strain, and several waves of exposure to the pathogen may be necessary to stimulate a strong neutralizing response; (b) as it has already proved for other viruses, such as Lassa,^24^ neutralizing antibodies are not always elicited after vaccination or natural infection; in fact, other mechanisms of the immune system may be involved in the protection, such as the complement‐fixation reaction mediated by IgG_1_ and IgG_3_, antigen‐dependent cellular cytotoxicity and T‐cell responses. Samples that are not able to show a high signal on ELISA (borderline samples) may, instead, have neutralizing capabilities, as it was confirmed by three of our samples. In this study, we show a possible and objective method of read‐out using spectrophotometry and a solution containing 0.02% of neutral red able to stain lysosomes and other cell organelles.^25^ Moreover, the aforementioned method increases throughput by enabling more samples to be processed per run. The difference between the titres registered by the two analysts in evaluation of CPE may be attributed to those wells where the ratio between the percentage of infected and uninfected cells is quite difficult to estimate under the microscope. The colorimetric method, on the other hand, based on a numerical value of optical density, obviates this problem. However, the present study has limitations. At this stage, the major difficulty lies in the lack of a standardized positive control that would enable the proper standardization of the assays. Furthermore, the number of samples analyzed in this preliminary assessment was small. The next step in this study will be to fully validate the colorimetric MN assay according to the criteria established by the International Council for Harmonization of Technical Requirements for Pharmaceuticals for Human Use.^26^ This will involve the inclusion of samples from individuals with confirmed SARS‐CoV‐2 diagnosis and the use of additional positive sera from other alpha or beta CoVs to investigate possible serological cross‐ reactions. Finally, another aspect to examine is the optimal infective dose to be use in the MN assay (100 TCID50 or lower) for this viral strain, to have a more reliable and accurate response based on the actual immunological status.

## 5 CONCLUSIONS

To conclude, the method of viral growth, titration and neutralization of SARS‐CoV‐2 presented in this study results suitable for the quantification of the neutralizing antibody titre in serum samples. Together with ELISA assay, this test should always be included in seroepidemiological and immunogenicity studies of vaccines. The necessity for a BSL 3 laboratory could certainly be a limiting factor for neutralizing antibodies studies using wild type viruses, but it is currently the most reliable method in terms of results provided.

## CONFLICT OF INTERESTS

The authors declare that there are no conflict of interests.

## AUTHOR CONTRIBUTIONS

AM designed and performed the neutralization set‐up experiments, conducted the viral growth procedures in BSL 3, and prepared the manuscript; MM and EC performed the neutralization tests in BSL 3 and elaborated the results; DM prepared and cultured the VERO E6 cell line. AT purchased the virus, the monoclonal antibody and cell lines; CMT and SM performed the ELISA assay at the University of Siena; EM supervised the study. All authors have approved the final version of the manuscript.
